# Clinical implication of ZEB-1 and E-cadherin expression in hepatocellular carcinoma (HCC)

**DOI:** 10.1186/1471-2407-13-572

**Published:** 2013-12-05

**Authors:** Motoyuki Hashiguchi, Shinichi Ueno, Masahiko Sakoda, Satoshi Iino, Kiyokazu Hiwatashi, Koji Minami, Kei Ando, Yuko Mataki, Kosei Maemura, Hiroyuki Shinchi, Sumiya Ishigami, Shoji Natsugoe

**Affiliations:** 1 Department of Digestive Surgery, Breast and Thyroid Surgery, Kagoshima University Graduate School of Medicine and Dental Sciences, Kagoshima, Japan; 2 Department of Clinical Oncology, Course of Advanced Therapeutics, Kagoshima University Graduate School of Medicine and Dental Sciences, Kagoshima, Japan

**Keywords:** Hepatocellular carcinoma, Hepatic resection, ZEB-1, E-cadherin, EMT

## Abstract

**Background:**

While recent research has shown that expression of ZEB-1 in a variety of tumors has a crucial impact on patient survival, there is little information regarding ZEB-1 expression in hepatocellular carcinoma (HCC). This study investigated the co-expression of ZEB-1 and E-cadherin in HCC by immunohistochemistry and evaluated its association with clinical factors, including patient prognosis.

**Methods:**

A total of 108 patients with primary HCC treated by curative hepatectomy were enrolled. ZEB-1 expression was immunohistochemically categorized as positive if at least 1% cancer cells exhibited nuclear staining. E-cadherin expression was divided into preserved and reduced expression groups and correlations between ZEB-1 and E-cadherin expression and clinical factors were then evaluated.

**Results:**

With respect to ZEB-1 expression, 23 patients were classified into the positive group and 85 into the negative group. Reduced E-cadherin expression was seen in 44 patients and preserved expression in the remaining 64 patients. ZEB-1 positivity was significantly associated with reduced expression of E-cadherin (p = 0.027). Moreover, significant associations were found between ZEB-1 expression and venous invasion and TNM stage. ZEB-1 positivity was associated with poorer prognosis (p = 0.025). Reduced E-cadherin expression was significantly associated with intrahepatic metastasis and poorer prognosis (p = 0.047). In particular, patients with both ZEB-1 positivity and reduced E-cadherin expression had a poorer prognosis (p = 0.005). Regardless of E-cadherin status, ZEB-1 was not a significant prognostic factor by multivariate analysis. There was no statistical difference in overall survival when E-cadherin expression was reduced in the ZEB-1 positive group (p = 0.24).

**Conclusions:**

Positive ZEB-1 expression and loss of E-cadherin expression are correlated with poor prognosis in HCC patients and malignancy of ZEB-1 positive tumors involves EMT.

## Background

Hepatocellular carcinoma (HCC) is a major health problem worldwide, with an estimated incidence ranging between 500,000 and 1,000,000 new cases annually. It is the fifth most common cancer in the world, and the third most common cause of cancer-related death. The disease is highly lethal because of its aggressive metastasis and an advanced stage at the time of diagnosis [1]. Recent developments in surgical and medical therapies have significantly improved the outcome of patients with both operable and advanced HCC [2,3]. Although there is recent evidence that these patients benefit from new molecular targeted therapies, systemic chemotherapy is not as effective as expected in patients with advanced HCC [4].

It has recently become clear that epithelial-mesenchymal transition (EMT) plays an important role in cancer progression, metastasis and chemoresistance, most likely involving a common molecular mechanism. However, the involvement of EMT varies greatly among cancer types, and much remains to be elucidated [5,6]. A hallmark of EMT is down-regulation of the cell adhesion molecule E-cadherin, a transmembrane protein essential for the establishment of stable adherent junctions, and up-regulation of mesenchymal molecules including vimentin, fibronectin and/or N-cadherin. It has been reported that repression of E-cadherin is associated with dedifferentiation, infiltrative growth and high incidence of lymph node metastasis in several cancers [7-9]. E-cadherin is repressed by multiple mechanisms, including gene mutation, promoter hypermethylation, or promoter repression by transcription repressors during tumor progression. A variety of transcription factors including the zinc finger Snail homologues (Snail1, Snail2/Slug, and Snail3) and several basic helix-loop-helix factors such as Twist, ZEB-1, and ZEB2, all interact with the E-box element within the proximal region of the E-cadherin promoter [5,8,10,11]. ZEB-1, like other EMT-inducing transcription factors such as Twist, Snail, Slug and SIP, binds DNA using similar E-box sequence motifs, thereby effecting repression of E-cadherin [12]. Aberrant expression of ZEB-1 in endometrial cancers, colorectal carcinomas and prostate cancer has been associated with aggressive disease, poor differentiation, the development of metastases and poor clinical prognosis [6,8-10].

In the oncogenic pathway, transforming growth factor-β (TGF-β) signaling is also critical for EMT induction [13]. The relationship between TGF-β and cancer promotion has been examined from various viewpoints [13-16], and recently, it has been reported that TGF-β stimulates EMT by two mechanisms [14]. The first, namely canonical signaling, involves a heterocomplex of activated Smad2/3 and smad4. The second, termed noncanonical signaling, involves induction of EMT gene expression by ZEB-1 and other transcription factors such as Snail, Twist or and Stat3, culminating in prolonged induction of EMT. We previously observed elevated expression of Smad4 in 35.5% of patients in HCC, and that this status was correlated with a poor prognosis [17].

The aim of this study was to investigate the association between the expression status of ZEB-1 and E-cadherin in HCC using immunohistochemistry, and to evaluate the clinical impact of the expression status of these proteins.

## Methods

### Patients and tumor samples

108 patients with primary single nodular HCC (85 men and 23 women, with a mean age of 65.3 years) were treated by hepatic partial resection between January 1996 and December 2002. Surgical specimens from these patients were used in this study. As shown in Table 1, of these 108 patients, 18 patients were positive for the hepatitis B surface antigen, 76 were positive for anti-hepatitis C virus antibodies, 1 was positive for both viruses and 13 were negative for both viruses. Mean tumor diameter was 44.3 mm (range 10–150 mm). The histological grade of each tumor and the tumor staging were determined by the General Rules for the Clinical and Pathological Study of Primary Liver Cancer (The Liver Cancer Study Group of Japan, 2009, 5th edition). 18 tumors (16.7%) showed well-differentiated HCC, 78 (72.2%) tumors were moderately differentiated, and 12 (11.1%) tumors were poorly differentiated. Follow-up data after surgery were obtained from all patients, with a median follow-up period of 48.4 months. Before tissue acquisition, each patient provided written informed consent to participate in the study, which was approved by the ethics committees of Kagoshima University School of Medicine.

**Table 1:** Characteristics of patients

**Gender**		
Male	85	(78.7%)
female	23	(21.3%)
Mean age	65.3 years	
Hepatitis virus type		
B	18	(16.7%)
C	76	(70.4%)
B + C	1	(0.9%)
None	13	(12%)
Mean tumor size	44.3 mm	
Histological grade (Differentiation)		
Well	18	(16.7%)
Moderate	78	(72.2%)
Poor	12	(11.1%)
Total	108	

### Antibodies

Goat anti-human polyclonal antibody to ZEB-1 was purchased from SANTA CRUZ BIOTECHNOLOGY, Inc. (Santa Cruz, CA, USA). Mouse anti-human monoclonal antibody to E-cadherin was purchased from DAKO JAPAN (Tokyo, Japan).

### Immunohistochemistry

Avidin-biotinylated peroxidase complex (ABC) immunohistochemistry was performed as follows. 4-mm thick sections were cut from paraffin blocks of HCC. After deparaffinization and rehydration, heat-induced antigen retrieval by autoclave pretreatment (120°C for 10 min) in citrate buffer solution (pH 6.0) was performed. Endogenous peroxidase activity was blocked by immersing the slides in absolute methanol solution containing 3% hydrogen peroxide for 10 min. Endogenous biotin activity was blocked using an avidin/biotin blocking kit purchased from NICHIREI, (Tokyo, Japan). Sections were incubated in avidin solution for 15 minutes followed a by brief rinse in PBS, after which sections were incubated in biotin solution for 15 minutes (all at room temperature). Sections were then treated with 1% bovine serum albumin for 30 min to block nonspecific reactions, after which they were incubated with ZEB-1 antibody (1:100 dilution) or E-cadherin antibody (1:100 dilution) for one hour at room temperature. Following incubation, specimens were visualized with an ABC detection kit (Vector laboratory, Burlingame, CA) and a diaminobenzidine (DAB) substrate system, according to the instructions provided by the manufacturer. Slides were counterstained with hematoxylin before mounting. All reactions were performed using appropriate positive and negative controls, and no significant staining was observed in the negative control sections.

### Evaluation of immunohistochemistry

In order to evaluate the results by immunohistochemical staining, ten fields of each specimen were selected. The expression in 1,000 tumor cells (100 cells/field) was evaluated with high-power (×400) microscopy. Two investigators (M.H. and S.U.) assessed the slides without knowledge of the clinicopathological features and were blinded to each other’s evaluation. They were in agreement on all the slides examined.

### Statistical analysis

Statistical analysis of group differences was performed using the χ2 test or Student’s t-test. The Kaplan-Meier method and subsequent evaluation by log-rank test were used for overall survival analysis. The prognostic factors were examined by univariate and multivariate analyses (proportional hazards regression model). A P-value of less than 0.05 was considered to be statistically significant.

## Results

### Expression of ZEB-1 and E-cadherin in HCC

**Figure 1:**
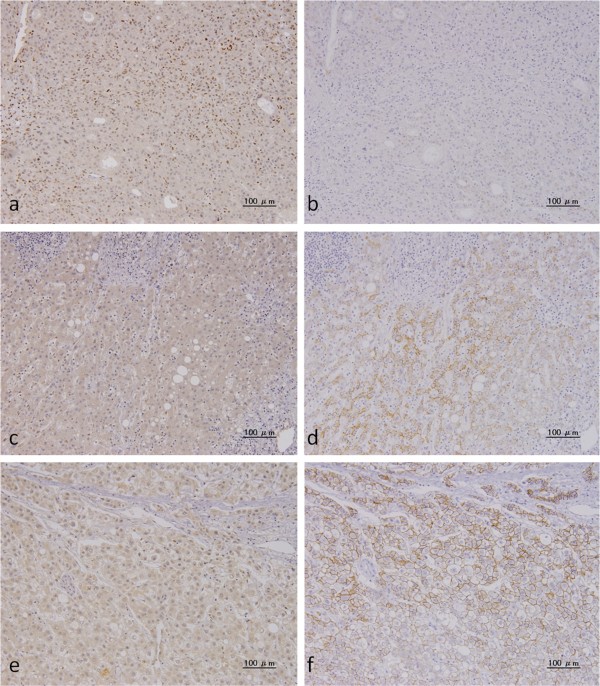
**Immunohistochemical analysis of ZEB-1 and E-cadherin expression.** ZEB-1 antibody was purchased from SANTA CRUZ BIOTECHNOLOGY, Inc. and E-cadherin antibody was purchased from DAKO JAPAN. Images from representative cases are shown: case 1 **(a - d)** and case 2 **(e. f)**. Case 1 was classified as >10% ZEB-1 positive. **a.** Positive expression of ZEB-1 in cellular nuclei in HCC. **b.** Reduced expression of E-cadherin in HCC cells. **c.** ZEB-1 expression is undetectable in noncancerous liver cells. **d.** E-cadherin expression was observed in the cell membrane in noncancerous liver cells. Case 2 was classified as ZEB-1 negative. **e.** ZEB-1 was not detected in the cell nuclei in HCC cells. **f.** E-cadherin expression was preserved in the cell membrane in HCC cells.

ZEB-1 was detected in the cellular nuclei of HCC cells (Figure 1a). All noncancerous liver cells were ZEB-1 negative (Figure 1c). ZEB-1 expression was classified into four groups: absent (n = 85; Figure 1e), 1 ~ 5% of all cancer cells (n = 12), 6 ~ 10% (n = 7) and >10% (n = 4). Since the frequency of ZEB-1 expression was low, ZEB-1 expression was categorized as positive if at least 1% of cancer cells exhibited nuclear staining (n = 23; 21.3%) or as negative if very few or no cancer cells were stained (n = 85; 78.7%).

E-cadherin was detected in the cellular membranes of HCC and in the normal glands of the liver (Figure 1d, f). E-cadherin expression was compared between malignant cells and noncancerous liver cells located away from the tumor. Tumor cells with a staining intensity equal to or greater than that of noncancerous liver cells were considered to be preserved expression (n = 64, 59.3%; Figure 1f)), whereas those with a weaker staining intensity than noncancerous liver cells or with no expression at all, were considered to be reduced expression (n = 44, 40.7%; Figure 1b).

### Correlation between ZEB-1 and E-cadherin expression and clinicopathological factors

Table 2 shows the correlation between immunohistochemical expression and clinicopathological factors. Positive expression of ZEB-1 in 23 HCCs (21.3%) was significantly associated with vascular invasion (p = 0.016) and advanced tumor TNM stage (p = 0.023). Furthermore, there was a trend towards an increased frequency of intrahepatic metastasis in the ZEB-1 positive group (p = 0.078). Reduced E-cadherin expression in 44 HCCs (40.7%) was significantly associated with intrahepatic metastasis (p < 0.001) and advanced tumor stage (p = 0.05).

**Table 2:** Clinicopathological Variables and ZEB1 and E-cadherin expression in HCC

		**ZEB1**			**E-cadherin**		
**Variable**	**Total no. (n = 108)**	**positive (n = 23)**	**negative (n = 85)**	***p*-value**	**preserved (n = 64)**	**reduced (n = 44)**	***p*-value**
Gender							
Male	85	18	67	0.996	47	38	0.107
Female	23	5	18		17	6	
Tumor size (mm)							
≧4.5 cm	33	10	27	0.227	21	16	0.702
<4.5 cm	75	13	58		43	28	
Vascular invasion							
Present	38	13	25	0.016	21	17	0.533
Absent	70	10	60		43	27	
Infiltration into capsule (Fc-inf)							
Present	84	18	66	0.95	51	33	0.565
Absent	24	5	19		13	11	
Intrahepatic metastasis							
Present	27	9	18	0.078	13	33	<0.001
Absent	81	14	67		51	11	
Gross classification^*^							
Localized	69	13	56	0.095	43	26	0.389
Invasive	29	10	19		21	18	
Differentiation^*^							
Well	18	3	15	0.5	9	9	0.717
Moderate	76	15	61		46	30	
Poor	12	4	8		7	5	
PIVKA II level (mAU/ml) (n = 91)							
Normal (≦40 )	23	5	18	0.906	16	7	0.36
High (>40 )	68	14	54		40	28	
AFP level (ng/ml) (n = 95)							
Normal (≦20 )	48	10	38	0.837	29	19	0.732
High (>20 )	47	9	38		30	17	
Pathological TMN Stage^*^							
I + II + III	88	15	73	0.023	56	32	0.05
IV	20	8	12		8	12	
Immunohistochemical staining							
ZEB-1							0.027
Positive	23		9		14		
negative	80				55	30	

^*^The histological grade of each tumor and the tumor staging were determined by the General Rules for the Clinical and Pathological Study of Primary Liver Cancer (The Liver Cancer Study Group of Japan, 2009, 5th edition).

Finally, reduced E-cadherin expression was significantly associated with positive ZEB-1 expression (p = 0.027).

### Prognostic impact of ZEB-1 and E-cadherin expression

**Figure 2:**
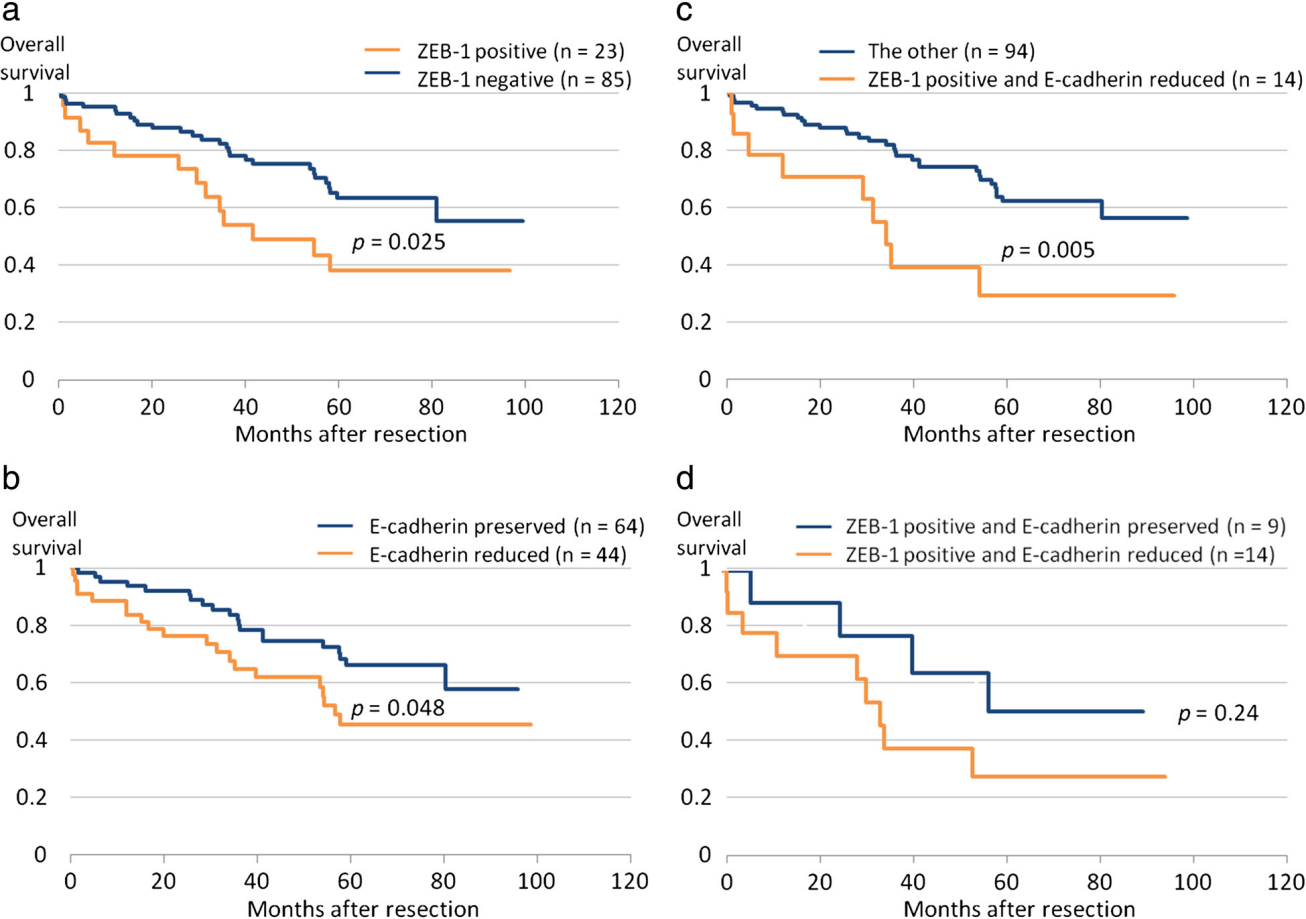
**Overall survival curves for each immunohistochemical staining group. a.** The 5-year survival rate was significantly lower in the ZEB-1 positive group than in the ZEB-1 negative group (5-year 38.1 vs. 63.4%, p = 0.025). **b.** The 5-year survival rate was significantly higher in the preserved E-cadherin group than in the reduced E-cadherin group (5-year 66.0 vs. 45.5%, p = 0.048). **c.** When comparing between patients with ZEB-1 positive/ E-cadherin reduced and patients with other expression pattern combinations, ZEB-1(+)/E-cadherin(-) group showed a significantly poorer prognosis (5-year 29.5 vs. 62.2%, p = 0.005). **d.** There was no statistical difference in overall survival when E-cadherin expression was reduced in the ZEB-1 positive group (p = 0.24).

Figure 2a & b shows overall survival curves after surgery according to ZEB-1 and E-cadherin expression (Figure 2a, b). The 5-year survival rates of patients with positive and negative expression of ZEB-1 were 38.1 and 63.4%, respectively (p = 0.025). Similarly, the 5-year survival rate was significantly better in the E-cadherin preserved group than in the reduced E-cadherin group (5-year 66.0 vs. 45.5%, p = 0.048).

Overall survival was evaluated according to the various combinations of the expression patterns of ZEB-1 and E-cadherin. When comparing between patients with ZEB-1 positive and reduced E-cadherin expression and patients with other expression pattern combinations (Figure 2c), the former group showed a significantly poorer prognosis (5-year 29.5 vs. 62.2%, p = 0.005).

There was no statistical difference in overall survival when E-cadherin expression was reduced in the ZEB-1 positive group (p = 0.24) (Figure 2d).

### Univariate and multivariate analyses

Factors relating to the patients’ prognosis were evaluated by univariate and multivariate analyses (Table 3). Univariate analysis showed that intrahepatic metastasis (p = 0.0007), vascular invasion (p = 0.047) and ZEB-1 expression (p = 0.037) were significantly related to postoperative survival. There was a strong trend towards association of reduced E-cadherin expression with poor prognosis (p = 0.053). In the multivariate analysis, only intrahepatic metastasis (p = 0.0086) was an independent prognostic factor.

**Table 3:** Univariate and Multivariate analysis of overall survival

	**Univariate**			**Multivariate**		
**Variables**	**HR**	**95%CI**	**p-value**	**HR**	**96%CI**	**p-value**
Intrahepatic metastasis	1.80	1.30-2.47	0.0007	1.67	1.14-2.41	0.0086
Vascular invasion	0.72	1.00-1.88	0.047	1.08	0.74-1.55	0.696
Positive ZEB1	1.45	1.02-2.00	0.037	1.20	0.83-1.71	0.320
Reducing E-cadherin	1.36	1.00-1.86	0.053	1.31	0.95-1.81	0.101

## Discussion

ZEB-1 (also known as dEF1, Nil-2-a, Tcf8, Bzp, Areb6, Meb1, Zfhx1a and Zfhep) has been identified as a nuclear factor that specifically binds to and represses the avian lens-specific d1-crystallin enhancer [18]. ZEB-1 is a DNA binding transcriptional repressor that interacts in a ligand-dependent fashion with receptor-activated Smad transcription factors involved in mediating TGF-β signaling [19]. Recent research has shown that expression of ZEB-1 has a crucial impact on patient survival [20]. Positive expression of ZEB-1 in endometrial cancers, colorectal carcinomas, and prostate cancer has been associated with aggressive disease, poor differentiation, development of metastases, and poor clinical prognosis [21-24]. In contrast, there is little information regarding the clinical implications of ZEB-1 expression in HCC, nor the relationship between ZEB-1 and E-cadherin expression in HCC.

In this study, we immunohistochemically investigated ZEB-1 expression in HCC and evaluated its association with clinical factors, including patient prognosis. For the purposes of this study, ZEB-1 positive expression was defined as >1% ZEB-1 positive HCC cells, although it should be noted that this is not an established method. A similar ZEB-1 positive percentage (14/110, 12%) in HCC has been previously reported in an immunohistochemical analysis [25]. We showed that positive expression of ZEB-1 was significantly associated with vascular invasion (p = 0.016), tumor TNM stage (p = 0.024), and prognosis (p = 0.025). Using western blot, Zhou et al. showed that elevated expression of ZEB-1 occurred in 65.4% (72/110) of HCC tissues and was a significant prognostic factor for poor overall and disease-free survival rates [26]. A caveat of Western blot is that it may not completely exclude the influence of differences in expression in different regions of the tumor, nor the contribution of contaminating fibroblasts. In order to better understand its effect on tumor function, ZEB-1 expression in HCC cells should be studied in isolation. Accordingly, in the present study we examined ZEB-1 expression using immunohistochemical analysis. In this report, while the frequency of ZEB1 expression in the nuclei of HCC cells was lower than that in previous reports [26], patient prognosis was significantly poorer when ZEB-1 positive cells were present in HCC tissues. Moreover, increased ZEB-1 expression cells was associated with markedly worse prognosis (data not shown). We suggest that immunohistochemical evaluation of ZEB-1 expression in tumor nuclei may have clinical prognostic impact.

Reduced E-cadherin expression has been observed in HCC, in particular in poorly-differentiated cancers [27,28]. We also showed that reduced expression of E-cadherin was significantly associated with increased intrahepatic metastasis (p < 0.001) and poorer prognosis (p = 0.048). When we analyzed the relationship between ZEB-1 and E-cadherin expression, the combination of positive ZEB-1 expression and reduced E-cadherin expression was associated with the worst prognosis among the various combinations of ZEB-1 and E-cadherin expression. Again using western blot, Zhou et al. also observed a significant correlation between lower E-cadherin protein expression and elevated ZEB-1 expression in HCC specimens [26]. High expression of ZEB-1 may further enhance its inhibition of the expression of the E-cadherin gene, causing a decrease in E-cadherin levels and an increase in migration and invasiveness in cancer cells [21-24,29,30]. Acquisition of an invasive phenotype through EMT, which enables cancer cells to break away from the primary tumor and invade surrounding tissues, may strongly promote the spread of cancer cells into the portal venous circulation.

During the EMT process, epithelial cancer cells acquire fibroblast-like properties and malignant potential through both canonical and non-canonical TGF-β signaling pathways [13,14]. Activation of canonical Smad2/3 signaling results in nuclear translocation of these factors with Smad4 and subsequent regulation of gene expression through their numerous interactions with additional transcriptional activators and repressors. We have previously shown that strong expression of Smad4 occurs in 35.5% of patients in HCC, and is associated with a poor prognosis (p = 0.04) [17]. In this study however, we found that a combination of ZEB1 and E-cadherin expression was a more powerful statistical tool than Smad4 in predicting clinical outcome. Alternatively, activation of factors in noncanonical TGF-β signaling, such as MAP kinases, small GTPases, PI3K/AKT, and NF-κB, also couples TGF-β to regulation of EMT expression programs. Finally, activation of transcription factors belonging to the Snail family (e.g., Snail, Twist, or ZEB-1), or of Stat3, induces genes associated with EMT, which ultimately promotes progression of EMT. In this study, we correlated positive expression of ZEB-1 and reduced expression of E-cadherin with poorer prognosis in HCC. We speculate that EMT is active in ZEB-1 positive tumors, and that both canonical and noncanonical signaling systems are influential in HCC.

## Conclusions

In conclusion, it is likely that positive ZEB-1 expression and reduced E-cadherin expression are correlated with the progression of HCC through their influence on the progression of EMT. Accordingly, inhibition of the expression or function of EMT-inducing transcription factors in malignant carcinoma is anticipated to lead to new therapeutic strategies.

## Competing interests

The authors declare that they have no competing interests.

## Authors’ contributions

MH and SU initiated the study, participated in its design and coordination, carried out the study, performed the statistical analysis. MH, SU and SN drafted the manuscript. MS, SI, KH, KM, KA, YM, KM, HS and SI provided data, contributed to data interpretation. All authors read and approved the final manuscript.

## Pre-publication history

The pre-publication history for this paper can be accessed here:

http://www.biomedcentral.com/1471-2407/13/572/prepub
